# Bepirovirsen induces innate immune proteins independent of HBV

**DOI:** 10.1007/s12072-026-11134-z

**Published:** 2026-08-04

**Authors:** Jared L. Delahaye, Shihyun You, Kai Fu, Scott Van Buren, Cheng Zhu, William Jordan, Avijit Ray, Johannes M. Freudenberg, Kristen M. Consalvo, Luca Pagliaroli, Sohil Joshi, Puneet Dhawan, Dickens Theodore, Melanie Paff, Jill Walker, Jennifer M. Singh

**Affiliations:** 1 HBV/Virology Translational Science, GSK, Collegeville, PA USA; 2 HBV Research, Collegeville, PA USA; 3 Translational Omics, GSK, Collegeville, PA USA; 4 Translational Omics, GSK, Waltham, MA USA; 5 Cellular Biomarkers, GSK, Collegeville, PA USA; 6 Clinical Development, GSK, Durham, NC USA; 7 Medical Development, GSK, Collegeville, PA USA; 8 HBV/Virology Translational Science, GSK, San Francisco, CA USA

**Keywords:** Phase 1 study, Phase 2a study, Biomarker analysis, Clustering analysis, Mechanism of action, Immune activation, Unconjugated antisense oligonucleotide, Soluble biomarkers, HBV, Chronic hepatitis B, Innate immunity

## Abstract

**Background:**

Bepirovirsen, an antisense oligonucleotide that targets all hepatitis B virus (HBV) mRNAs, is in development for the treatment of chronic HBV infection. Clinical studies have demonstrated that bepirovirsen has multiple mechanisms of action, including lowering HBV DNA, reducing viral protein production, and inducing immune activation. Post hoc analysis of biomarker samples from three studies was performed to further characterize changes in soluble protein biomarkers after bepirovirsen dosing.

**Methods:**

Across three studies (CS3 [NCT02981602] in participants with chronic HBV infection; PFS [NCT06058390] and QTc [NCT06422767] in healthy volunteers) participants received bepirovirsen (150 mg, 300 mg, or 450 mg) or placebo. Longitudinal serum samples were taken for exploratory proteomics analysis. Relative expression of soluble proteins, including immune-related biomarkers, was measured, and differential expression was determined across arms. Clustering analysis was performed to identify kinetic patterns.

**Results:**

Bepirovirsen induced significant, transient protein differential expression in the hours after dosing, while there were relatively minor changes in placebo. There were many similarities in protein biomarkers induced in CS3 participants with chronic HBV infection and in the two healthy volunteer studies. Additionally, bepirovirsen treatment increased abundance of many proteins to a higher degree with increasing dose.

**Conclusions:**

These findings strengthen the association between bepirovirsen treatment and changes in innate immune-related proteins and indicate that this effect does not require the presence of HBV. The kinetic patterns of protein expression suggest that there are multiple waves of protein induction, which could impact recruitment and activation of innate and adaptive immune cell types.

**Clinical trial number:**

NCT02981602, NCT06058390, NCT06422767.

**Graphical Abstract:**

Bepirovirsen induces proteomic changes consistent with a transient innate immune response in healthy volunteers and participants with chronic HBV infection

**Figure:**
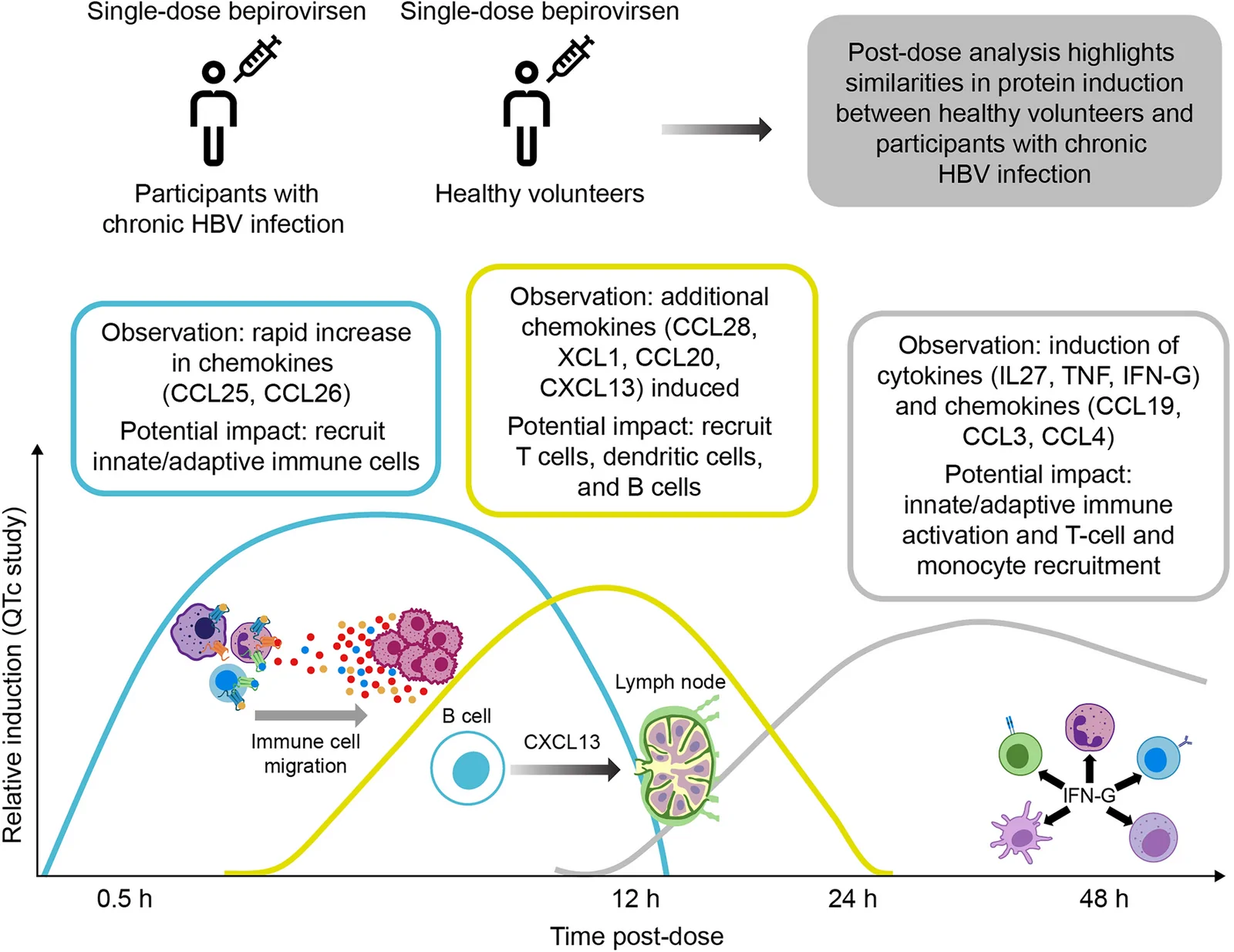

**Supplementary Information:**

The online version contains supplementary material available at https://doi.org/10.1007/s12072-026-11134-z.

## Introduction

Chronic hepatitis B virus (HBV) infection is a major global health issue [1]. The persistence of chronic HBV infection results from HBV replication and the production of viral proteins, such as hepatitis B surface antigen (HBsAg), which in turn promote immune dysfunction [2]. Addressing all three of these factors (HBV replication, viral protein production, and immune response) is critical for achieving functional cure, defined as at least 24 weeks of a sustained HBV DNA level below the lower limit of quantification and HBsAg loss after finite (fixed-duration) therapy, with or without a positive test for hepatitis B surface antibody [3,4].

Bepirovirsen is an investigational unconjugated antisense oligonucleotide that has completed Phase 3 trials for the treatment of chronic HBV infection with functional cure as the treatment goal [5]. It is a single-stranded nucleic acid that binds to and targets all HBV mRNAs, thus reducing HBsAg levels and suppressing viral DNA [6,7]. In addition to measuring antiviral activity, multiple chronic HBV infection Phase 2 studies have assessed the effect of bepirovirsen treatment on immune biomarkers, including its impact on B-cell proliferation and T-cell activation. However, these analyses lacked acute timepoints [8–12].

Here, a post hoc analysis from three independent studies was conducted to further characterize the effect of bepirovirsen treatment on biomarkers and its role in immune stimulation at early timepoints post-dosing.

## Materials and methods

This analysis included data from three studies (QTc study, NCT06422767; prefilled syringe [PFS] study, NCT06058390; and CS3 study, NCT02981602), described below. Biomarker samples, which contributed to the objectives of this exploratory analysis, were collected with consideration for the clinical and operational constraints specific to each study.

### Study designs

#### QTc study

**Fig. 1:**
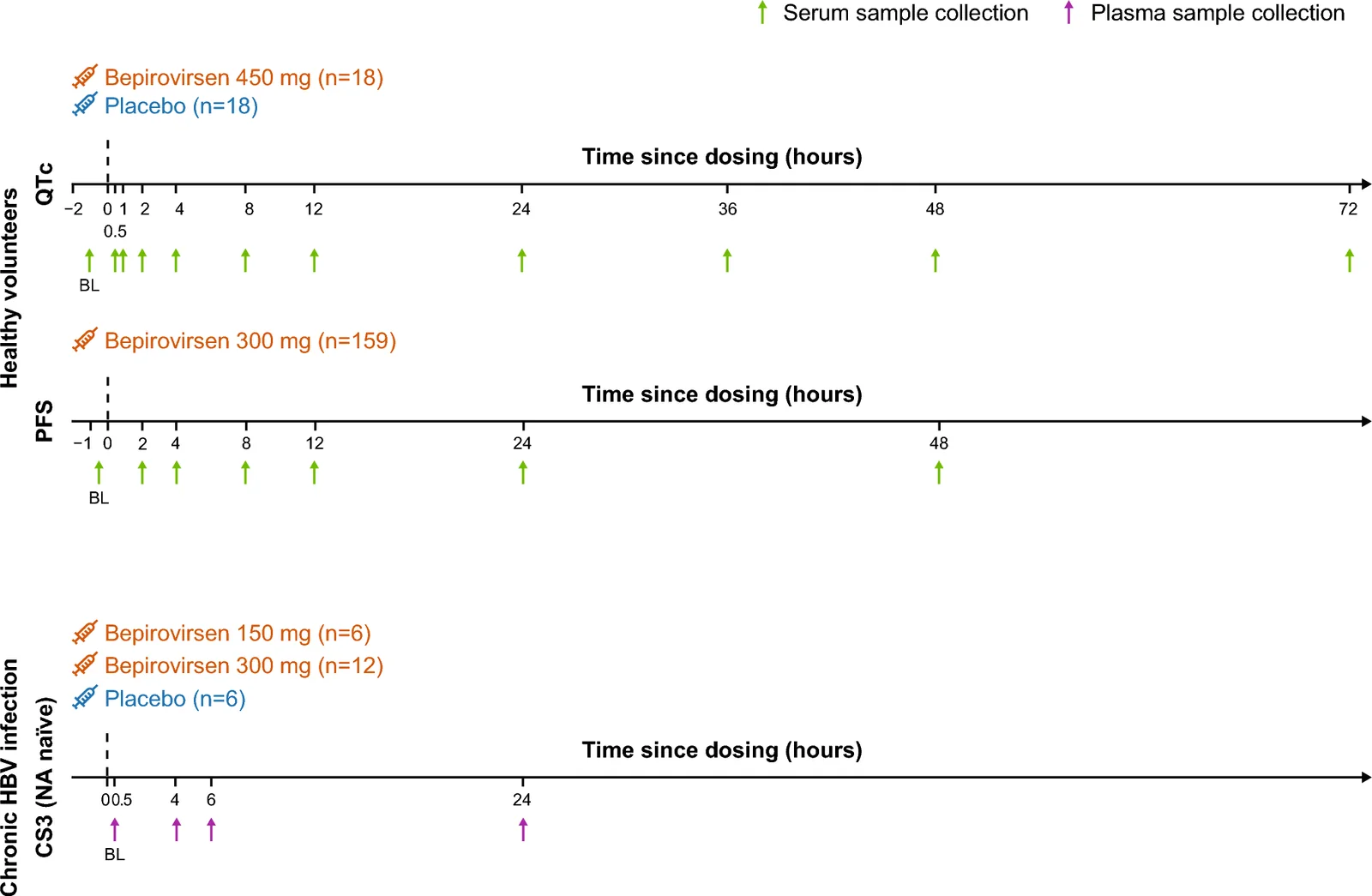
Study design. *BL* baseline, *HBV* hepatitis B virus, *NA* nucleos(t)ide analog, *PFS* prefilled syringe

The QTc study was a Phase 1, randomized, double-blind, placebo-controlled, parallel-group study designed to evaluate the effect of bepirovirsen on cardiac conduction, pharmacokinetics, safety, and tolerability. Healthy volunteers were randomized to receive a single supratherapeutic subcutaneous dose of bepirovirsen (450 or 600 mg) or placebo (3 × 1 mL or 4 × 1 mL normal saline). Participants receiving bepirovirsen 450 mg (n = 18) or placebo 3 × 1 mL (n = 18) were included in this analysis. Serum samples were collected at 11 timepoints: baseline (within 2 h prior to dosing), 0.5, 1, 2, 4, 8, 12, 24, 36, 48, and 72 h post-dose (Fig. 1).

#### PFS study

The Phase 1 PFS study design, inclusion criteria, and demographics have been described previously [13]. Briefly, healthy volunteers were randomized (3:3:2:2) to receive a single 300 mg dose of bepirovirsen administered by a healthcare professional (HCP) from a vial (Group 1) or a ready-to-use PFS assembled with a safety syringe device (Group 2) or self-administered using the PFS with (Group 3) or without (Group 4) training from an HCP. The randomization schedule also included different injection sites (thigh, abdomen, and upper arm). Data from participants who received at least one dose of study intervention (n = 159) were included in this analysis. Serum samples were collected at 7 timepoints: baseline (within 1 h prior to dosing), 2, 4, 8, 12, 24, and 48 h post-dose (Fig. 1).

#### CS3 study

The Phase 2a CS3 study design, inclusion criteria, demographics, and baseline characteristics have been described previously [6]. Briefly, participants with chronic HBV infection who were either treatment-naïve or receiving stable nucleos(t)ide analog (NA) therapy were randomized (3:1) to receive bepirovirsen (treatment-naïve: 150 mg or 300 mg; NA treated: 300 mg) or placebo (normal saline) administered via subcutaneous injection on Days 1, 4, 8, 11, 15, and 22. Data from treatment-naïve participants (bepirovirsen 150 mg, n = 6; bepirovirsen 300 mg, n = 12; placebo, n = 6) were used in this analysis. For acute timepoint analysis, plasma samples were collected at 0.5 (baseline), 4, 6, and 24 h post-dose (Fig. 1).

### Protein expression data generation and preprocessing

Serum or plasma proteomic profiling was performed using the Olink Explore 1536 platform (Olink Proteomics, Uppsala, Sweden), which measures 1,472 unique proteins, as per manufacturer’s protocol. The respective protein expression profiles were represented as normalized protein expression (NPX) values, which measure relative protein abundance on the log_2_ scale. In addition to NPX values, the limit of detection (LOD) and quality check (QC) warning flags were reported by the sample analysis laboratory. For sample quality control, principal component analysis (PCA) was conducted to detect any outlier samples exhibiting distinct patterns compared with other samples. In addition, PCA plots were visually inspected to ensure samples with QC warnings did not show large separation from other samples. All samples passed QC and were retained for the final analysis. Protein assays with NPX values greater than the LOD in ≤ 20% of samples were excluded due to low expression. These proteins showed no association with treatment group or timepoint, such that their removal did not bias downstream results. Additionally, the three proteins (TNF, CXCL8, IL8) shared as common overlapping assays across the four panels of the Olink Explore 1536 platform were averaged across the four assays to yield a single measurement per protein per sample. Serum proteomic profiling was also performed using the Olink Target 48 platform for the QTc study, measuring the absolute concentrations (pg/mL) of 45 unique cytokines. Similar QC checks were performed for the Olink Target 48 platform data as previously described for the Olink Explore 1536 platform.

### Protein data analysis

Differential expression analysis was conducted to compare NPX values (Olink Explore 1536 platform) or log_2_ protein concentrations (Olink Target 48 platform) at baseline to subsequent timepoints after dosing within each study. Statistical modeling was conducted using the linear models for microarray data (limma) method [14], which uses random effects to account for the correlation in the repeated measurements from the same participants across time. Covariates included in the PFS study modeling were body mass index (BMI), injection site, sex, and timepoint, with timepoint by sex interactions additionally included when appropriate. Subgroup analysis by randomized group (administration by HCP or self, vial or PFS) was initially considered, but ultimately, groups were pooled due to the lack of systematic differences. In the QTc study, variables included in the model were treatment group and timepoint. Other covariates were not included due to the lower study size. The Benjamini–Hochberg method was applied to calculate adjusted p-values to account for multiple hypothesis testing [15]. Log_2_ fold change from limma was used to represent changes from baseline at each post-dosing timepoint. To determine the optimal number of protein clusters, the elbow method was applied, evaluating the within-cluster sum of squares (WSS) for k values ranging from 1 to 10. The optimal k value was selected at the point where further increases resulted in only minimal reductions in WSS. In the CS3 study, a generalized linear mixed effects model was fitted to compare changes in protein expression from baseline at each timepoint. For each analyte at each timepoint, R-emmeans (v1.5.3) was used to estimate if mean log_2_ fold change was significantly different from baseline.

### Integrated analysis

To assess the consistency of analyte changes across independent studies and the impact of bepirovirsen dose on protein levels, average NPX changes from baseline were compared between the CS3, PFS, and QTc studies. It should be noted that treatment effects were not directly compared across studies. Instead, treatment effects were first assessed relative to baseline within each study, and the resulting fold changes were subsequently compared across studies. To confirm the validity of this approach, the fold changes within the bepirovirsen 300 mg groups of the CS3 and PFS studies were compared and found to be highly correlated, despite the methodological differences between the studies. Median values of the log_2_ fold change ratio (median ratio) were calculated to represent the overall difference in protein log_2_ fold change between studies.

## Results

### Comparative proteomic analysis between healthy volunteers and participants with chronic HBV infection

**Fig. 2:**
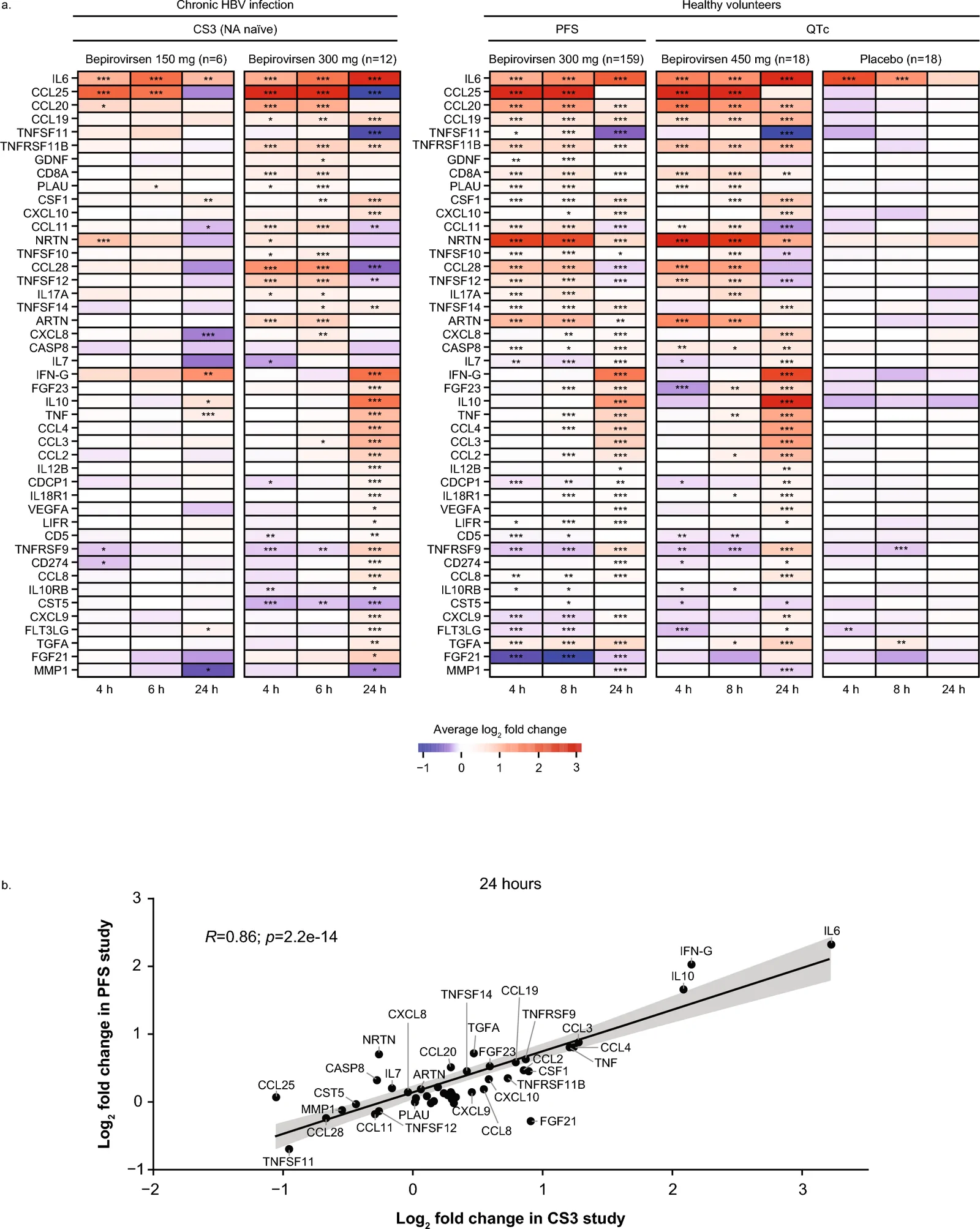
**a** Comparison of protein fold changes from baseline at 4, 6–8, and 24 h post-bepirovirsen dose between the CS3, PFS, and QTc studies. **b** Representative correlation plot of protein fold changes at 24 h post-dose between participants receiving 300 mg bepirovirsen in the CS3 and PFS studies. Panel a: Adjusted p-values, *p < 0.05; **p < 0.01; ***p < 0.001. Panel b: Correlation plots at 4 and 6–8 h post-dose are shown in Fig. S1. *HBV* hepatitis B virus; *NA* nucleos(t)ide analog; *PFS* prefilled syringe

In the CS3 study, participants with chronic HBV infection were treated with bepirovirsen and proteomics was performed after a single dose. Preliminary analysis with a smaller multiplex platform identified a set of proteins that were differentially expressed at or before 24 h post-dose [16,17]. Given that this was a relatively small study, this protein list, which includes many immune-related proteins, was used to create heatmaps (using the Olink Explore 1536 platform) showing the within study change from baseline for the CS3, QTc, and PFS studies, focusing on the similar timepoints up to 24 h post-dose to evaluate the consistency in immune modulation across these independent studies (Fig. 2a). In general, the proteins that showed changes in expression after bepirovirsen treatment in the CS3 study (bepirovirsen 150 mg, n = 6; bepirovirsen 300 mg, n = 12) also showed induction in the participants treated with bepirovirsen in the PFS (n = 159) and QTc (n = 18) studies. Notably, many of the proteins in the top half of the list were up-regulated between 4 and 8 h after treatment with bepirovirsen, while many in the bottom half were up-regulated at 24 h. In contrast, very few proteins were differentially expressed at any timepoint in the placebo group, indicating that these changes in protein expression were largely the effect of bepirovirsen treatment. In addition, correlation analysis was performed for the bepirovirsen 300 mg dose groups from the CS3 and PFS studies. This showed a high correlation in the degree of protein induction in participants receiving the same dose of bepirovirsen who either had chronic HBV infection or were healthy volunteers (Fig. 2b, Fig. S1). Together, this demonstrates that in two independent healthy volunteer studies, many similar proteins are up-regulated when compared to those seen in participants with chronic HBV infection. This supports that the changes in immune-related proteins induced by bepirovirsen does not require interaction with HBV viral components.

### Effects of bepirovirsen dose on protein expression

**Fig. 3:**
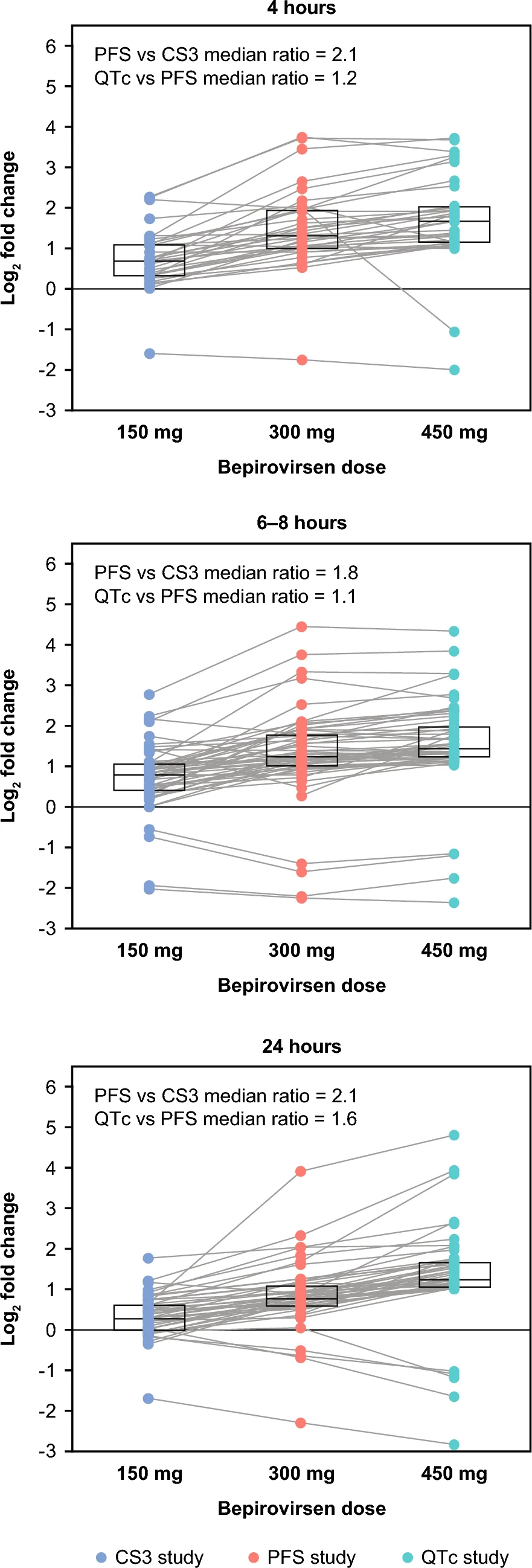
Protein induction across CS3, PFS, and QTc studies increases with dose. The log_2_ fold change for the differentially expressed proteins identified in the QTc study are shown for the three studies. In the PFS and QTc studies an 8 h timepoint was used while a 6 h timepoint was used in CS3. Median ratio: ratio (division) of median values of log_2_ fold change. *PFS* prefilled syringe

To evaluate the impact of bepirovirsen dose, differential protein expression analysis was performed using the QTc bepirovirsen 450 mg dose group to ensure proteins that may not have changed significantly at lower doses were captured. The log_2_ fold change from baseline of all differentially expressed proteins was compared with the timepoints that overlapped between the PFS and CS3 studies (Fig. 3). Generally, proteins that were positively induced (> 0 log_2_ fold change from baseline) had a greater fold change in the group that received the higher bepirovirsen dose (450 mg vs 300 mg vs 150 mg). This observation would require follow-up in other studies to understand if there is a formal dose–response relationship.

### Differential protein expression and clustering

**Fig. 4:**
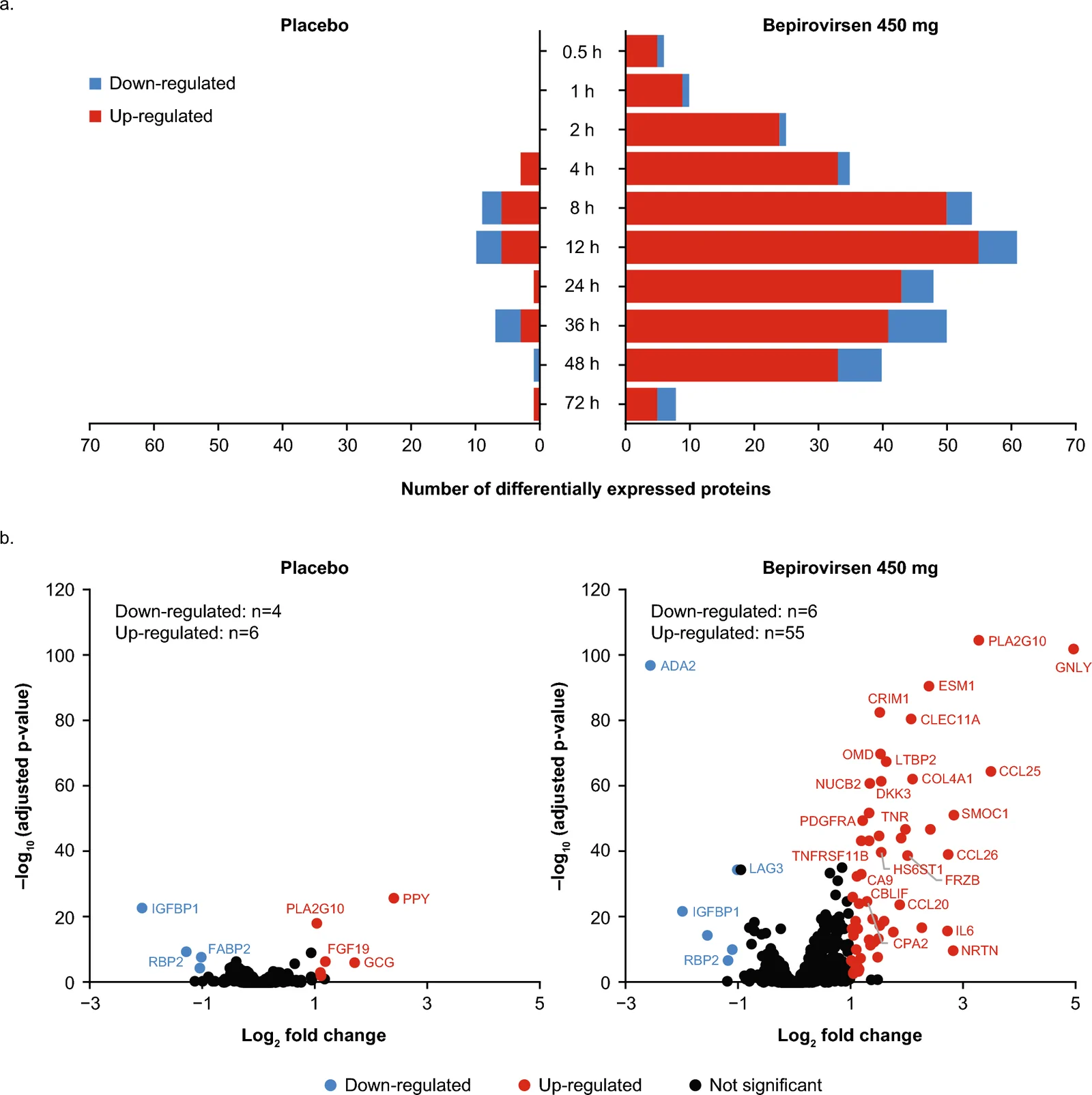
Differentially expressed proteins in participants receiving bepirovirsen 450 mg or placebo by timepoint (QTc study). **a** Number of differentially expressed proteins from baseline by timepoint. **b** Representative volcano plots from the 12 h timepoint. Panel b: Proteins significantly up-regulated from baseline are shown in red; proteins significantly down-regulated from baseline are shown in blue. Names of some of the differentially expressed proteins are shown as examples. Significance cut-off: absolute log_2_ fold change > 1 and adjusted p-value < 0.05. Additional volcano plots are shown in Fig. S2

To better characterise temporal changes across all soluble proteins measured, the QTc dataset was utilized due to having the largest range of timepoints post-dose. The differential expression analysis performed in the previous section (in the bepirovirsen 450 mg dose group) was extended to the QTc study participants who received placebo (Fig. 4). The bepirovirsen group had more than 100 significantly differentially expressed proteins across the time course, while there were relatively few differentially expressed proteins in the placebo group (Fig. 4a). The volcano plots of each of the timepoints highlight the number of significantly up-regulated and down-regulated proteins (Fig. 4b, Fig. S2). Some proteins were differentially expressed as early as the 0.5 h timepoint, while others were up or down-regulated later. The highest number of differentially expressed proteins occurred at the 12 h timepoint.

**Fig. 5:**
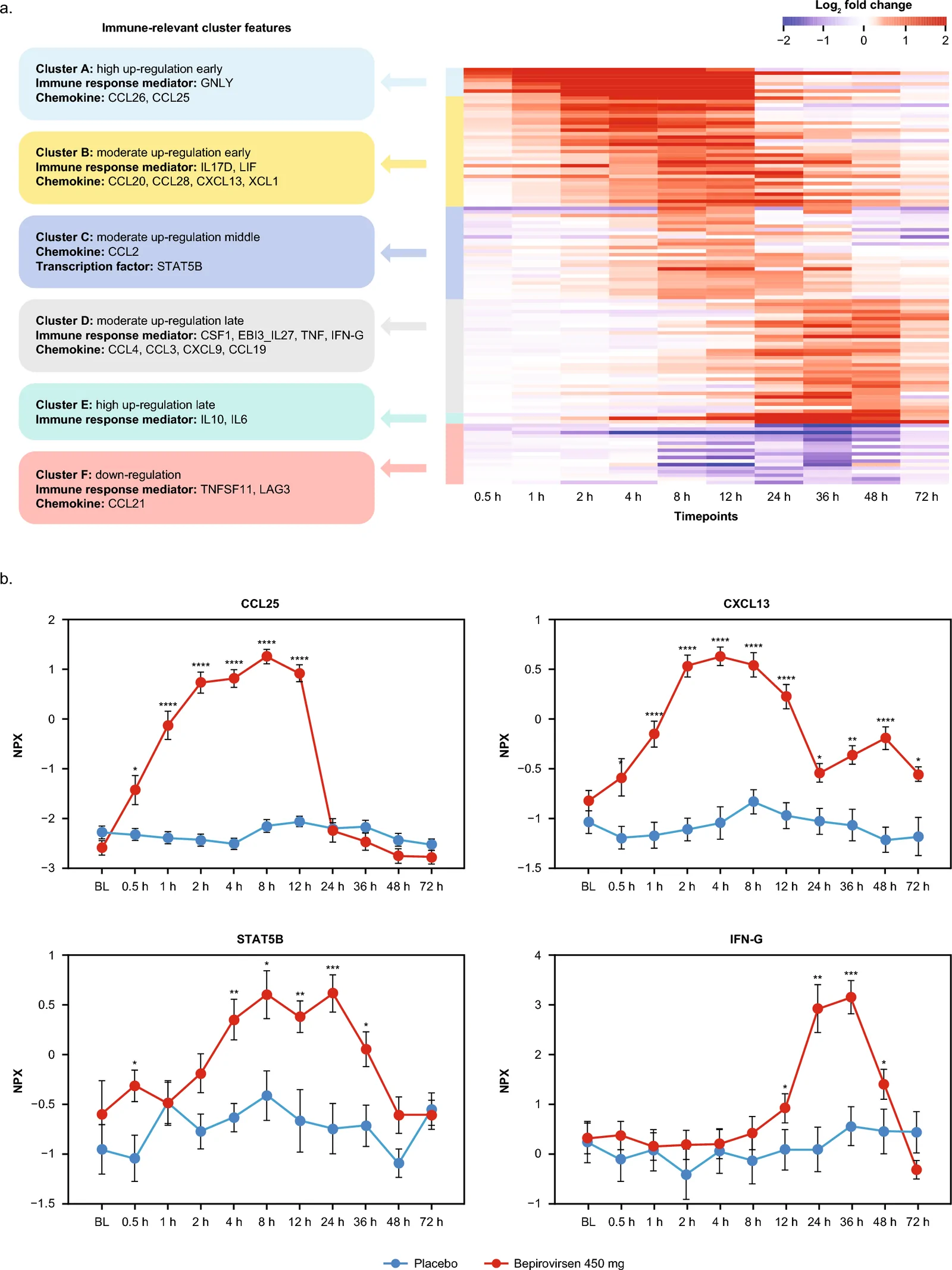
Cluster analysis from participants receiving bepirovirsen 450 mg (QTc study). **a** Heatmap showing change from baseline in differentially expressed proteins. **b** Representative line plots of analytes induced with varying kinetics. Panel a: The full list of proteins identified from the cluster analysis are listed in Table S1. Panel b: Data are mean ± SE. Adjusted p-values, *p < 0.05; **p < 0.01; ***p < 0.001; ****p < 0.0001. *BL* baseline, *NPX* normalized protein expression, *SE* standard error

Exploratory K-means clustering analysis revealed groups of up- or down-regulated proteins with similar longitudinal expression profiles, some of which were induced rapidly, and others that peaked more than 24 h after dosing (Fig. 5a). The complete list of all differentially expressed proteins is shown in Table S1, and a specific review of the protein lists from the clusters that were up-regulated with varying kinetics showed that these contained multiple factors with known roles in mediating immune responses (Fig. 5a). Representative line plots of analytes from some of the clusters are shown in Fig. 5b. Cluster A, which peaked the earliest, included the chemokines CCL25 and CCL26. Cluster B was also up-regulated early, peaking by 8 h, and included the additional chemokines CXCL13, XCL1, CCL20, and CCL28. Cluster C peaked slightly later, tended to have a slightly lower induction, and included the chemokine CCL2 and the transcription factor STAT5B. Finally, cluster D peaked starting at 24 h and included the cytokines IFN-G, TNF, and IL27 (also referred to as EBI3_IL27) as well as the chemokines CCL3, CCL4, CCL19, and CXCL9. These temporal patterns, with earlier induction (< 24 h) of proteins including CCL25 and later induction (≥ 24 h) of other proteins including IFN-G, are consistent with the CS3 and PFS studies. Quantitative analysis using an orthogonal proteomics approach, the Olink Target 48 platform, was also performed (**Fig. S3**) and generally demonstrated similar kinetic patterns for analytes that overlapped with those shown in Fig. 2 and 5. Representative line plots show expression of CCL2, which peaked around 12–24 h, and TNF, which peaked around 24–36 h.

Due to data availability limitations, the previous CS3 study analysis (Fig. 2) left open the question of when does protein induction resolve. By capturing later timepoints uniquely available in the QTc study, it appears that this effect is transient. Most of the differentially expressed proteins peaked at or prior to 48 h and subsequently decreased at or before the 72 h timepoint.

## Discussion

Through the course of clinical development of bepirovirsen, the antiviral effects of HBsAg reduction and HBV DNA suppression have been well characterized [6,7]. While progress has been made to examine the additional immunostimulatory mechanism of bepirovirsen [8, 17], this has been challenging due in part to the complexity of sampling at the most relevant timepoints. Here, we have been able to reinforce and expand upon our understanding of bepirovirsen’s acute immune effects through strategic biomarker sampling in three independent studies to address relevant questions concerning the effects of HBV, dose, and kinetics.

Analysis of the set of proteins previously identified to be induced acutely in the CS3 study (participants with chronic HBV infection) [17] generally showed similar induction in the QTc and PFS studies (healthy volunteers). This shows that there is a direct effect of bepirovirsen on the immune system that occurs regardless of the presence or impact of HBV viral components. In addition, the majority of differentially expressed proteins identified from the QTc study showed higher induction from 150 mg (CS3 study) to 300 mg (PFS study) to 450 mg (QTc study) when an integrated analysis was performed over all of the studies.

It was encouraging to see that the proteins first identified in the small CS3 study generally seemed to have similar changes in the other two healthy volunteer studies. However, in order to more broadly investigate the proteomic response, the QTc study was used because it had the greatest number of timepoints going out to 72 h post-dose. Investigation of the QTc study biomarker time course demonstrated that administration of bepirovirsen 450 mg led to significant differential protein expression with varying kinetic patterns over the 72 h post-dose, while there were relatively minor changes in the placebo group. Within the first 2 h post-dose, there was a rapid increase in proteins, including chemokines (CCL25, CCL26) that could contribute to the recruitment of innate/adaptive immune cells [18]. There was also an increase in a separate group of analytes which peaked by 8 h. This group included additional chemokines capable of T cell (CCL28), dendritic cell (XCL1) and B cell (CCL20 and CXCL13) recruitment [18,19]. Finally, starting at 24 h post-bepirovirsen treatment, another wave of analytes began increasing. This included cytokines (IL27, TNF, IFN-G) capable of activating a broad range of innate/adaptive immune cells (including B cells, T cells, macrophages, dendritic cells, and NK cells) [20]. It also contained chemokines, including those that could support T-cell (CCL19, CXCL9) or monocyte recruitment (CCL3, CCL4) [21,22]. Importantly, these proteomic changes consistent with early immune activation appear to be transient, based on the attenuation of expression by the 72 h timepoint, which is when the second loading dose of bepirovirsen was given in the Phase 3 study [5]. Together, these data suggest that bepirovirsen induced dynamic changes in innate immune markers that occurred in multiple waves.

These analyses were exploratory in nature, and the three clinical studies were not formally powered for these endpoints. As such, while we believe they provide valuable insights and support hypothesis generation, they should be interpreted in light of some limitations. Between the three studies, there were a number of methodological differences, including the sample size, sample matrix, and timepoints, which precluded more rigorous statistical analyses across studies. It is also important to recognize that the scope of biomarker analysis in these studies was restricted to proteomics. Thus, the interplay of how changes in these proteins may impact cellular recruitment and activation would need to be addressed via additional approaches.

In conclusion, this proteomics analysis from three independent studies strengthens our understanding of bepirovirsen's immune activation mechanism. The impact of this early immune activation on repeat dosing with bepirovirsen, HBV-specific adaptive immune responses, antiviral effects, and clinical outcomes requires further investigation in larger studies of participants with chronic HBV infection.

## Supplementary Information

Below is the link to the electronic supplementary material.

Supplementary file1 (DOCX 741 KB)

## Data Availability

Please refer to GSK’s weblink to access GSK’s data sharing policies and as applicable seek anonymized subject level data via the link https://www.gsk-studyregister.com/en/.
