# Supplementary material for: Bepirovirsen induces innate immune proteins independent of HBV
Source: Hepatol Int. 2026 Aug 4;20(5):1318–28. doi: 10.1007/s12072-026-11134-z (PMC13676563; doi:10.1007/s12072-026-11134-z)
Supplement: Supplementary file 1 [file 12072_2026_11134_MOESM1_ESM.docx]

**Bepirovirsen induces innate immune proteins independent of HBV**

Jared L. Delahaye PhD^1^, Shihyun You PhD^2^, Kai Fu PhD^3^, Scott Van Buren PhD^4^, Cheng Zhu PhD^5^, William Jordan PhD^6^, Avijit Ray PhD^7^, Johannes M. Freudenberg PhD^8^, Kristen M. Consalvo PhD^9^, Luca Pagliaroli PhD^10^, Sohil Joshi MS^11^, Puneet Dhawan PhD^12^, Dickens Theodore MD^13^, Melanie Paff PhD^14^, Jill Walker PhD^15^, Jennifer M. Singh PhD^16^

^1^HBV/Virology Translational Science, GSK, Collegeville, PA, USA, [jared.l.delahaye@gsk.com](mailto:jared.l.delahaye@gsk.com), [+16109175000]; ^2^HBV Research, Collegeville, PA, USA, [shihyun.k.you@gsk.com](mailto:shihyun.k.you@gsk.com), [+19172578586]; ^3^Translational Omics, GSK, Collegeville, PA, USA, [kai.8.fu@gsk.com](mailto:kai.8.fu@gsk.com), [+16109175000]; ^4^Translational Omics, GSK, Collegeville, PA, USA, [scott.k.vanburen@gsk.com](mailto:scott.k.vanburen@gsk.com), [+16109175000]; ^5^Translational Omics, GSK, Waltham, MA, USA, [cheng.8.zhu@gsk.com](mailto:cheng.8.zhu@gsk.com), [+15086549851]; ^6^Translational Omics, GSK, Collegeville, PA, USA, [william.t.jordan@gsk.com](mailto:william.t.jordan@gsk.com), [+16109175000]; ^7^HBV Research, Collegeville, PA, USA, [avijit.r.ray@gsk.com](mailto:avijit.r.ray@gsk.com), [+18329713810]; ^8^Translational Omics, GSK, Collegeville, PA, USA, [johannes.m.freudenberg@gsk.com](mailto:johannes.m.freudenberg@gsk.com), [+16109175000]; ^9^Cellular Biomarkers Genomics, GSK, Collegeville, PA, USA, [kristen.m.consalvo@gsk.com](mailto:kristen.m.consalvo@gsk.com), [+16109175000]; ^10^Cellular Biomarkers Genomics, GSK, Collegeville, PA, USA, [luca.x.pagliaroli@gsk.com](mailto:luca.x.pagliaroli@gsk.com), [+16109175000]; ^11^Cellular Biomarkers Genomics, GSK, Collegeville, PA, USA, [sohil.x.joshi@gsk.com](mailto:sohil.x.joshi@gsk.com), [+16109175000]; ^12^Cellular Biomarkers, GSK, Collegeville, PA, USA, [puneet.x.dhawan@gsk.com](mailto:puneet.x.dhawan@gsk.com), [+16109175000]; ^13^Clinical Development, GSK, Durham, NC, USA, [dickens.x.theodore@gsk.com](mailto:dickens.x.theodore@gsk.com), [+19194830727]; ^14^Medical Development, GSK, Collegeville, PA, USA, [melanie.t.paff@gsk.com](mailto:melanie.t.paff@gsk.com), [+19194833573]; ^15^HBV/Virology Translational Science, GSK, San Francisco, CA, USA, [jill.t.walker@gsk.com](mailto:jill.t.walker@gsk.com), [+19197459158]; ^16^HBV/Virology Translational Science, GSK, Collegeville, PA, USA, [jennifer.m.singh@gsk.com](mailto:jennifer.m.singh@gsk.com), [+16109175000]

**Corresponding author:**

Name: Jared L. Delahaye

Address: HBV/Virology Translational Science, GSK, Collegeville, PA, USA

Tel: +16109175000

Email: [jared.l.delahaye@gsk.com](mailto:jared.l.delahaye@gsk.com)

## Supplementary material

### Fig. S1. Correlation plots of protein fold changes at 4 and 6–8 hours post-dose between participants receiving 300 mg bepirovirsen in the CS3 and PFS studies

PFS, prefilled syringe.

### Fig. S2. Volcano plots showing differentially expressed proteins in participants receiving (a) bepirovirsen 450 mg or (b) placebo by timepoint (QTc study)

Proteins significantly up-regulated from baseline are shown in red; proteins significantly down-regulated from baseline are shown in blue. Names of some of the differentially expressed proteins are shown as examples. Significance cut-off: absolute log_2_ fold change >1 and adjusted p-value <0.05.

### Fig. S3. Quantitative analysis of differentially expressed proteins (QTc study). (a) Heatmap showing change from baseline in differentially expressed proteins for participants who received bepirovirsen 450 mg. (b) Representative line plots of analytes induced with varying kinetics in participants who received either placebo or bepirovirsen 450 mg

Panel a: Adjusted p-values, *p<0.05; **p<0.01; ***p<0.001. SE, standard error.

### Table S1. Protein lists from cluster analysis of differentially expressed proteins identified in participants receiving bepirovirsen 450 mg (QTc study)

| **Cluster A** | | | | | | | | | | | | |
| --- | --- | --- | --- | --- | --- | --- | --- | --- | --- | --- | --- | --- |
| HS6ST1 | GNLY | CCL26 | | CCL25 | | TFPI2 | | FRZB | | NRTN | | SMOC1 |
| **Cluster B** | | | | | | | | | | | | |
| ESM1 | NUCB2 | TIMP3 | DKK4 | | SOST | | IL17D | | ARTN | | CCL20 | |
| SFRP1 | NOS3 | TNFRSF11B | BGN | | CCL28 | | HS3ST3B1 | | CRIM1 | | CXCL13 | |
| LTBP2 | LIF | PLA2G10 | WASF3 | | KAZALD1 | | CLEC11A | | TP53 | | NPM1 | |
| PTX3 | DKK3 | XCL1 | SPP1 | | TNR | | COL4A1 | | OMD | |  | |
| **Cluster C** | | | | | | | | | | | | |
| GCG | FGF19 | RSPO1 | CBLIF | | KRT18 | | NCF2 | | TMPRSS15 | | CCL2 | |
| GH1 | CA9 | CPA2 | PAPPA | | ANXA10 | | STAT5B | | CLEC4D | | NMNAT1 | |
| FMNL1 | PPY | PDGFRA | CERT1 | | PFKFB2 | | NINJ1 | | RRM2B | | ANGPTL1 | |
| USO1 | PNLIPRP2 |  |  | |  | |  | |  | |  | |
| **Cluster D** | | | | | | | | | | | | |
| TNFRSF9 | VWF | CSF1 | EBI3_IL27 | | FCRLB | | TNFRSF13B | | CCL4 | | VWA1 | |
| SIGLEC5 | IGSF3 | MMP12 | FSTL3 | | CEACAM21 | | TNFRSF1B | | CCL3 | | IL1RN | |
| VCAM1 | MCFD2 | CTSB | FCER2 | | PTS | | IL18BP | | TNF | | CALCA | |
| TNFRSF6B | CXCL9 | CCL19 | IFN-G | | IL1RL1 | | SLAMF8 | | SCGB3A2 | | LBP | |
| **Cluster E** | | | | | | | | | | | | |
| IL10 | IL6 | PLA2G2A |  | |  | |  | |  | |  | |
| **Cluster F** | | | | | | | | | | | | |
| TNFSF11 | SERPINA12 | ADA2 | | CTSV | | EPHA1 | | FABP2 | | SLC39A5 | | FABP1 |
| EREG | RBP2 | CTSF | | IGFBP1 | | CCL21 | | LAG3 | | CTRC | | BANK1 |
| STX16 |  |  | |  | |  | |  | |  | |  |
